# Inhibitory effects of sodium pentosan polysulfate on formation and function of osteoclasts derived from canine bone marrow

**DOI:** 10.1186/s12917-018-1466-4

**Published:** 2018-05-02

**Authors:** H. M. Suranji Wijekoon, Eugene C. Bwalya, Jing Fang, Sangho Kim, Kenji Hosoya, Masahiro Okumura

**Affiliations:** Department of Veterinary Clinical Sciences, Laboratory of Veterinary Surgery, Graduate School of Veterinary Medicine, Hokkaido University, Sapporo, 060-0818 Japan

**Keywords:** Sodium pentosan polysulfate, Osteoclast differentiation, Bone marrow, Bone resorption, Dog

## Abstract

**Background:**

Sodium pentosan polysulfate (NaPPS) was testified as a chondroprotective drug in with a detailed rationale of the disease-modifying activity. This study was undertaken to determine whether anti-osteoarthritis drug, NaPPS inhibited osteoclasts (OC) differentiation and function. Canine bone marrow mononuclear cells (*n* = 6) were differentiated to OC by maintaining with receptor activator of nuclear factor kappa B ligand (RANKL) and macrophage colony-stimulating factor (M-CSF) for up to 7 days with the treatment of NaPPS at concentration of 0, 0.2, 1 and 5 μg/mL. Differentiation and function of OC were accessed using tartrate-resistant acid phosphate (TRAP) staining and bone resorption assay, while monitoring actin ring formation. Invasion and colocalization patterns of fluorescence-labeled NaPPS with transcribed gene in OC were monitored. Gene expression of OC for cathepsin K (CTK), matrix metallopeptidase-9 (MMP-9), nuclear factor of activated T-cells cytoplasmic 1 (NFATc1), c-Fos, activator protein-1(AP-1) and carbonic anhydrase II was examined using real-time PCR.

**Results:**

Significant inhibition of OC differentiation was evident at NaPPS concentration of 1 and 5 μg/mL (*p* < 0.05). In the presence of 0.2 to 5 μg/mL NaPPS, bone resorption was attenuated (*p* < 0.05), while 1 and 5 μg/mL NaPPS achieved significant reduction of actin ring formation. Intriguingly, fluorescence-labeled NaPPS invaded in to cytoplasm and nucleus while colocalizing with actively transcribed gene. Gene expression of CTK, MMP-9 and NFATc1 were significantly inhibited at 1 and 5 μg/mL (*p* < 0.05) of NaPPS whereas inhibition of c-Fos and AP-1 was identified only at concentration of 5 μg/mL (*p* < 0.05).

**Conclusions:**

Taken together, all the results suggest that NaPPS is a novel inhibitor of RANKL and M-CSF-induced CTK, MMP-9, NFATc1, c-Fos, AP-1 upregulation, OC differentiation and bone resorption which might be a beneficial for treatment of inflammatory joint diseases and other bone diseases associated with excessive bone resorption.

## Background

Bone homeostasis is crucial to maintain the integrity of bone functions that coordinates balance between bone resorption by osteoclasts (OC) and bone formation by chondrocytes/osteoblasts [1, 2]. It is well established that an imbalance in the function of OC and osteoblasts has severe consequences for the organism, leading to serious bone pathologies such as osteoporosis, joint and bone diseases involving the immune system including, rheumatoid arthritis (RA) and periodontal disease [3]. Osteoclasts, which originate from monocyte/macrophage lineage from bone marrow hematopoietic precursors [4] are the principal multinucleate giant resorptive cells of bone. Two major factors such as macrophage colony-stimulating factor (M-CSF) and receptor activator of NF kappa B ligand and (RANKL) are required for their differentiation and maturation [5–7]. Receptor activator of NF kappa B ligand binds to its receptor, receptor activator of NF kappa B on OC precursors [8], which involve the activation of NF-kB and Jun N-terminal kinase, eventually leading to the expression of activator protein-1 (AP1)/ Fos, an essential regulator of OC differentiation [9]. Previously reports have shown that OC precursors are found in both peripheral blood and synovial tissues of human with RA [10, 11]. Osteoclast precursors which are unable to resorb bone [12] stained positive for tartrate-resistant acid phosphatase (TRAP) eventually fuse to form multinucleated mature TRAP-positive OC [13]. Thus maturation and functional capability of OC while differentiation, are critical cellular process, understanding its regulation will have an important impact on the development of a new therapy to control bone loss among human and dogs.

In the past few years, the concept of disease -modifying osteoarthritis drugs (DMOADs) have been explored as an alternative treatment modality for osteoarthritis (OA) [14] instead of using nonsteroidal anti-inflammatory drugs (NSAIDs) which has been frequently used for treating OA and associate with a high risk for gastrointestinal lesions with long-term uses [15]. Sodium pentosan polysulfate (NaPPS) is a semi-synthetic sulfated polysaccharide drug manufactured from European beech-wood hemicellulose by sulfate esterification with the average molecular weight of 5700 Da [16]. From the results of previous in vitro and in vivo studies, the spectrum of pharmacological activities exhibited by NaPPS would qualify it as DMOADs [17] because of its ability to preserve the integrity of the articular cartilage and bone while improving the quality of the joint synovial fluid [18–21].

Although PPS has been used for a number of years for the treatment of thrombotic and hyperlipidemic indications [20] it has only recently been shown to be effective in improving the symptoms of human patients with OA [16]. While the molecular mechanism of PPS action at the microenvironment of joint remains unclear, some previous reports show that, NaPPS is capable in enhancing synthesis of proteoglycans such as aggrecan, which is intimately associated with resist compression throughout the extracellular matrix of articular cartilage [22]. Hence, synovial changes in dogs with canine arthritis mimic human RA, dogs are the potential useful model for studies of therapy [23]. Recently, it has been reported that NaPPS can inhibit osteogenic differentiation in human bone marrow derived precursor cells while inducing chondrogenic differentiation from bone marrow-derived mesenchymal stem cells in canine as well as human [24, 25]. Use of progenitor cells origin from different sources is being used for investigating the therapeutic effects and to imply its clinical uses [26]. However, among the several previous human and animal studies of NaPPS based on cartilage research, there was dearth of information on effect of NaPPS over the osteoclastogenesis, anti-resorptive capability and influence on cell signaling molecules of OC. To the best of our knowledge, present study is the first attempt to identify the interaction of NaPPS with in vitro cultured OC-derived from dog bone marrow. The objective of the study reported here was to determine whether there was an effect of NaPPS on osteoclastogenesis of canine bone marrow-derived hematopoietic precursor cells. We hypothesized that the NaPPS, which carry different effect by improving the symptoms of OA would more likely to have an inhibitory effect on OC differentiation and its signaling pathways.

## Methods

### Osteoclastic differentiation from canine bone marrow

Proximal femur of one year old, healthy beagle dogs (*n* = 6) were used to collect the 5 mL of bone marrow samples in to 10 mL syringe containing 1 mL Dulbecco’s modified eagle’s medium (DMEM, Life technologies, New York, USA) and 1000 U/mL of heparin (Nipro, Osaka, Japan). The use of all samples from healthy experimental dogs was in accordance with Hokkaido University Institutional Animal Care and Use Committee guidelines (approval number: 12–0059). Separation of bone marrow mononuclear cell (BMMs) fraction was done and preceded as described previously [27, 28]. Briefly, BMMs was obtained by density gradients centrifugation over lymphoprep (Axis-sheild PoC AS, Oslo, Norway) to remove red blood cells. Isolated BMMs cell fraction (5 × 10^6^ cells/mL) was incubated with DMEM containing penicillin/streptomycin (100 units/mL, Wako pure chemical, Tokyo, Japan) and 10% heat-inactivated fetal bovine serum (FBS, Nichirei Bioscience INC., Tokyo, Japan) for 24 h to separate the non-adherent and adherent cells. Non-adherent were collected as a source of immature OC precursors, suspended in DMEM, counted, seeded on 48-well plates (Corning, New York, USA) at 2 × 10^5^ cells/well, and cultured in DMEM with the presence of 20 ng/ml recombinant human M-CSF (Invitrogen, Maryland, USA) for 3 days. After 3 days, adherent cells were used as OC precursors after washing out the non-adherent cells, including lymphocytes and further cultured in the presence of 25 ng/mL M-CSF, 50 ng/mL recombinant human RANKL (Sigma-Aldrich, St Louis, Missouri, USA) to generate osteoclast-like multinucleated giant cells. The cells were treated with 0, 0.2, 1 and 5 μg/mL concentration of NaPPS (Cartrophen Vet-Biopharm-100 mg/ml, New South Wales, Australia) for 1-week. The selected concentrations of NaPPS are within the previous proved non-cytotoxic range for bone marrow derived cells [24]. Triplicate cultures for each concentration of NaPPS were maintained by changing the media in every 48 h ensuring their constancy of concentrations.

### Tartrate-resistant acid phosphate (TRAP) staining

Cultured BMMs with M-CSF and RANKL in the presence or absence of NaPPS were subjected to TRAP stain (Cosmo Bio Co., LTD, Tokyo, Japan) after 7 days. Cells were washed with 1% phosphate buffered saline (PBS) and fixed with 10% formalin neutral buffer solution for 5 min at room temperature. After washing with 500 μL deionized water 3 times, cells were stained for TRAP according to the manufacturer’s instructions. Cells containing ≥3 nuclei were considered as OC and counted.

### Pit formation assay

Non-adherent cells, collected from BMMs fraction of 3 dogs were cultured at 2 × 10^5^ cells/well density on bone resorption assay plate 48 (PG Research, Tokyo, Japan) which was coated with calcium phosphate (CaP-coated). The cells were maintained in DMEM with the presence of 20 ng/ml recombinant human M-CSF (Invitrogen, Maryland, USA) for 3 days in triplicate cultures. After 3 days, adherent cells were used as OC precursors after washing out the non-adherent cells and further cultured in the presence of 25 ng/mL M-CSF, 50 ng/mL recombinant human RANKL and NaPPS at various concentrations (0, 0.2, 1 and 5 μg/mL). After 7 days, the CaP-coated plate was treated with 5% sodium hypochlorite (Sigma-Aldrich, St Louis, Missouri, USA) for 5 min according to the manufacturer’s instructions. The resorption pit area was analyzed and counted by Image-J software (Image J software version 1.43, National Institute of Health).

### Actin ring formation assay

The actin ring formation assay was performed as described previously [29]. Briefly, BMMs cultured with M-CSF, RANKL and various concentrations of NaPPS for 7 days were washed with PBS and fixed with 4% paraformaldehyde (Wako pure chemical, Tokyo, Japan) in PBS on ice for 20 min. Osteoclasts were detergent-permeabilized with 0.2% Triton X-100 (ICN Biomedicals, Germany) in PBS for 10 min, washed and blocked in 10% normal goat serum (Sigma-Aldrich, St Louis, Missouri, USA) in PBS for 1 h. The cells were incubated with primary rabbit anti-F actin polyclonal antibody (Bioss Inc., Massachusetts, USA) (1:100 dilution) for 1 h in PBS with 1% normal goat serum, washing three times with PBS, incubating for 1 h with fluorescein isothiocyanate (FITC)-conjugated goat anti-rabbit antibody (Sigma) (1:100 dilution) in PBS with 1% normal goat serum, washing three times with PBS, and finally mounting with aqueous mounting medium. The images were observed by counting the number of actin rings under a laser scanning confocal microscope (Zeiss, Illinois, USA).

### Immunocytochemical detection of localization of NaPPS with actively transcribed gene

Osteoclast precursors resulting from canine bone marrow cells (2 × 10^5^ cells) were cultured in 8-well culture slide (Iwaki, Tokyo, Japan) in 400 μL of DMEM, 10% FBS with OC differentiation factors. Cells were incubated with 10 μg/mL of Tetramethylrhodamine (TRITC)-labeled NaPPS (Arthropharm, New South Wales, Australia) for 24 h. After fixation and blocking, cells were incubated with primary anti-human c-Jun (HT-9) rabbit polyclonal antibody (Santa Cruz Biotechnology, Dallas, Texas, USA) (1:100 dilution) in 1% normal goat serum followed by incubation with FITC-conjugated goat anti-rabbit antibody (Sigma) (1:100 dilution) in 1% normal goat serum. The OC were observed for detecting the colocalization patterns of NaPPS with transcribed gene (c-Jun) under a laser scanning confocal microscope.

### mRNA isolation and RT-PCR

Total RNA from cells was extracted using RNeasy Mini Kit (QIAGEN, Germantown, Maryland, USA) according to the manufacture’s protocol. Total RNA was quantified by spectrophotometry at 260 nm. RNA with a 260/280 nm ratio in the range 1.8–2.0 was considered high quality and then transcribed into cDNA with M-MLV RT kit (Takara Bio, Tokyo, Japan) according to manufacturer’s recommended procedures. One microgram of total RNA derived was reverse-transcribed into cDNA with random hexamers. PCR conditions were as follows: denaturation at 95 °C for 30 s, annealing temperature for 1 min, extension at 72 °C for 1 min for 30 cycles, and final extension at 72 °C for 7 min. PCR products were separated on 1.5% agarose gel (BM Equipment, Tokyo, Japan) and stained with ethidium bromide (Nippon Gene, Tokyo, Japan).

### Real-time PCR

Quantitative real-time PCR analysis was performed with KAPA SYBR® FAST qPCR kit (KAPA). The amount of 2 μL of cDNA template was added to each 10 μL of premixture with specific primers. The following primer sets were used: carbonic anhydrase II (CAII), 5’-AAGGAGCCCATCAGCGTTAG-3′ (forward) and 5’-GGGCGCCAGTTATCCATCAT-3′ (reverse); NFATc1, 5’-CACAGGCAAGACTGTCTCCA-3′ (forward) and 5’-TCCTCCCAATGTCTGTCTCC-3′ (reverse); MMP-9, 5’-GGCAAATTCCAGACCTTTGA-3′ (forward) and 5’-TACACGCGAGTGAAGGTGAG-3′ (reverse); c-Fos, 5′- GTCCGTACAGACCACAGACC-3′ (forward) and 5’-CGCTCCACTTCATTGTGCTG-3′ (reverse); CTK, 5′- ACCCATATGTGGGACAGGAT-3′ (forward) and 5’-TGGAAAGAGGTCAGGCTTGC-3′ (reverse); AP-1, 5’-TCTACGACGATGCCCTCAAC-3′ (forward) and 5’-TGAGCAGGTCCGAGTTCTTG-3′ (reverse); GAPGH, 5’-CTGAACGGGAAGCTCACTGG-3′ (forward) and 5’-CGATGCCTGCTTCACTACCT-3′ (reverse). All reactions were normalized to the housekeeping gene b-Actin. glyceraldehyde-3-phosphate dehydrogenase (GAPDH).

### Statistical analysis

Quantitative real-time PCR data, number of OC, resorption pits and actin rings were analyzed using SPSS software (SPSS software ver. 07 for Windows; SPSS Inc., Chicago, Illinois, USA). Analysis of variance (ANOVA) was used to compare the mean values between the treatments. Where significant different observed, multiple comparison of group means was performed using Post Hoc Bonferroni. Significant level was defined as *p* < 0.05. All quantitative results are presented as mean ± SE.

## Results

**Fig. 1:**
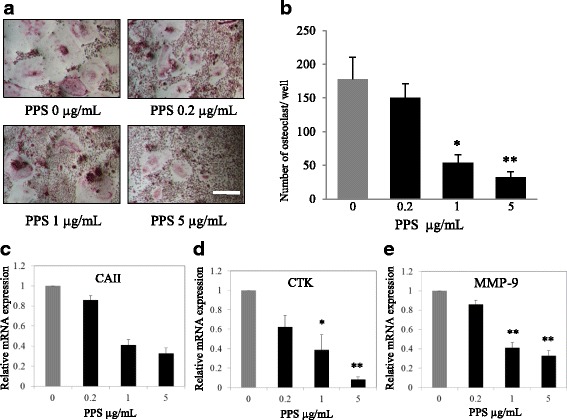
Shows inhibitory effect of NaPPS on canine OC differentiation. **a** The cells were treated with various concentration of NaPPS followed by M-CSF (20 ng/mL) and RANKL (50 ng/mL) for 7 days. The cells were stained for TRAP stain and **b** TRAP-positive cells (≥3 nuclei) were counted. Scale bar- 100 μm. Bar graphs show the concentration effects of NaPPS on mRNA expression levels of **c** CA II, **d** CTK and **e** MMP-9 determined by real-time PCR and results were normalized to the expression of GAPDH. Data are representative of five independent experiments and expressed as means ± SE. Means with *are significantly different from 0 μg/mL of NaPPS (*p < 0.05, **p < 0.01)

The effect of different concentration of NaPPS on OC differentiation from BMMs stimulated with RANKL and M-CSF was evaluated. The number of TRAP-positive multinuclear cells (≥3 nuclei) generated in 48 well plate were reduced with the administration of NaPPS at the concentration of 1 and 5 μg/mL NaPPS (*p* < 0.05) (Fig. 1a, b). Osteoclast specific genes, CAII, CTK and MMP-9 expression were analyzed after treatment of NaPPS at the concentration of 0.2, 1and 5 μg/mL. Relative mRNA expression level of CTK and MMP-9 genes were significantly downregulated (*p* < 0.05) at 1 and 5 μg/mL concentrations of NaPPS (Fig. 1c, d, e).

**Fig. 2:**
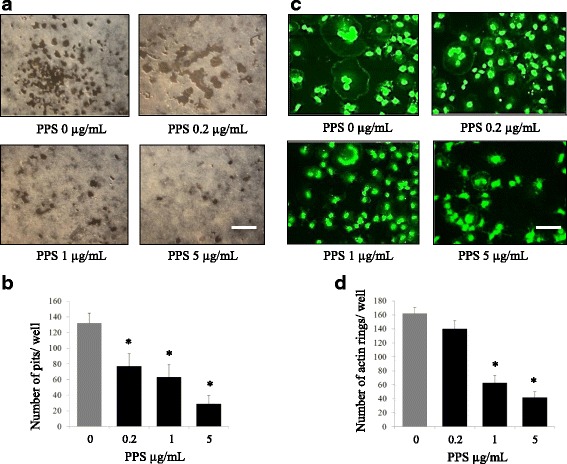
NaPPS inhibits bone resorption and actin ring formation by concentration gradient. Canine BMMs, cultured with M-CSF (25 ng/mL) and RANKL (50 ng/mL) for 7 days with or without indicated doses of NaPPS. **a** The cells were washed and the resorption pits were counted. **b** The numbers of pits were analyzed with Image-J software. **c** Cells were fixed, stained for F-actin formation (top) and **d** osteoclasts with actin ring were counted (bottom). Scale bar- 200 μm. Column indicates means ± SE of three experiments performed in triplicate. Means with *are significantly different from 0 μg/mL of NaPPS (*p < 0.05)

NaPPS on bone resorption was assessed with OC generated from 3 dogs. Cells were plated on CaP-coated plates and stimulated with M-CSF and RANKL in the presence or absence of NaPPS. Cells stimulated with M-CSF and RANKL formed a number of resorption pits (Fig. 2a, b), suggesting that the bone resorption activity of RANKL-treated cells made them into functionally active state resembling OC. All the concentrations of NaPPS (0.2, 1 and 5 μg/mL) significantly reduced the formation of resorption pits in number and in overall area compared with treatment with M-CSF and RANKL alone. In the presence of RANKL exposure, BMMs can differentiate into mature OC and form distinct actin-ring structures (Fig. 2c, d). However, NaPPS significantly reduced the number of actin-ring structures at 1 and 5 μg/mL concentrations, suggesting the inhibitory effect of certain concentration of NaPPS over the functional unit of OC.

**Fig. 3:**
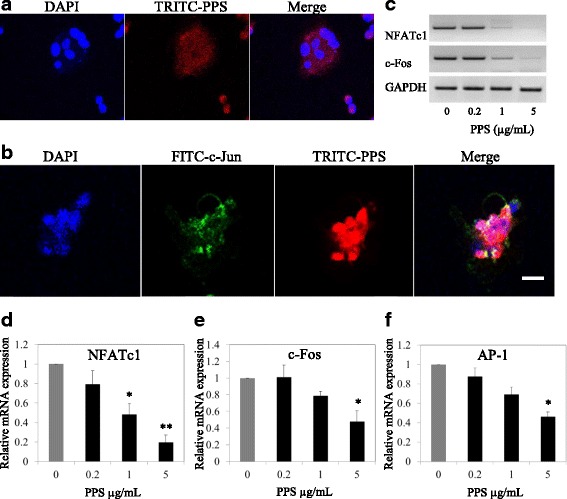
Inhibitory and co-localization patterns of NaPPS with actively transcribed genes. Osteoclast differentiated from canine bone marrow with supplement of M-CSF (25 ng/mL) RANKL (50 ng/mL) together with absence and presence of NaPPS (0.2, 1, 5 μg/mL) for 7 days was done. **a** Confocal microscopic images of invasion of TRITC-labeled NaPPS in to osteoclast and **b** localization with c-Jun (subunit of AP-1 transcription complex) are shown. The orange in the overlay image indicates that NaPPS and gene activation proteins c-Jun (green) are in the same location. Nuclei were stained with 4,6-diamidino-2-phenylindole dye (DAPI). Scale bar- 100 μm. **c** Attenuation effect of NaPPS on master regulators of osteoclastogenesis (NFATc1, c-Fos) by dose dependent pattern are shown. **d, e** and **f** Relative mRNA expression of NFATc1, c-Fos and AP-1 according to the NaPPS concentration gradient was significantly attenuated at 5 μg/mL. Data expressed as mean ± SE for each PPS concentration after normalizing for the expression of the GAPDH. Means with *are significantly different from 0 μg/mL of NaPPS (*p < 0.05, **p < 0.01)

Canine OC were treated with fluorescently labelled (red) pentosan polysulfate (TRITC-PPS), then fixed in formalin and fluorescently stained for genetic DNA (blue) and for c-Jun (green) which is one of the key components of AP-1 transcription factor [12]. Fluorescence-labeled NaPPS invaded in to cytoplasm and nucleus (Fig. 3a). The yellow to orange in the overlay image indicates that NaPPS and c-Jun proteins are in the same location (Fig. 3b). Pentosan inhibits the expression of NFATc1 at 1 and 5 μg/mL (*p* < 0.05) (Fig. 3d, e, f). And decrease c-Fos and AP-1 activation was identified only at concentration of 5 μg/mL (*p* < 0.05). As shown in Fig. 3c, semi quantitative RT-PCR was correlated with quantitative PCR.

## Discussion

In the present study, NaPPS at concentration of 5 μg/mL exerted an inhibitory effect on canine osteoclastogenesis through suppression of key transcription factors such as NFATc1, c-Fos while visualizing co-localization patterns. This information may partially support the suggestion that NaPPS may exert its inhibitory effect on OC by direct interaction with transcription factors, subsequently deterring the target genes like CTK and MMP-9 which are needed in bone resorption activity of OC. To further study the effects of NaPPS on osteoclastogenesis, we examined whether NaPPS affected RANKL-induced OC function by bone resorption assays and actin formation. The results suggested that NaPPS at concentration of 1 and 5 μg/mL suppressed RANKL-induced bone resorption activity and formation of actin-rings of matured OC. The stimulation of M-CSF and RANKL make mature OC result in resorption lacunae, pit formation and actin ring formation [30] which is a prerequisite for OC bone resorption and is the most obvious character of mature OC during osteoclastogenesis [31]. The outcome of this study suggests that the inhibitory action of NaPPS over OC differentiation and function could be applied in treatment of pathological bone disorders where OC play central role.

In this study, the noted inhibition of OC formation, TRAP activity and density of pits at 1 and 5 μg/mL of NaPPS indicated that an inhibitory effect on osteoclastogenesis and function of mature OC. The commonly used phenotype marker, TRAP is expressed particularly in OC and positive for TRAP stain after pre-OC cells differentiation with the supplement of RANKL [27]. Detection of TRAP-positive cell formation is a renowned method of determining OC formation and function [32, 33].

In our study, NaPPS at higher concentrations significantly suppressed the NFATc1 up-regulation in OC normally seen with RANKL treatment. In previous knock-out experiments have been demonstrated that NFATc1 [34, 35] and c-Fos are important transcription factors for RANKL-mediated OC differentiation, fusion, and activation [33]. In addition, previous reports demonstrated that NFATc1 is not induced by RANKL stimulation in OC lacking c-Fos [36]. Further, NFATc1 is the master regulator of osteoclastogenesis which is regulated by the AP-1 complex [37]. Dimeric transcription factor, AP-1 is composed of members of the Jun and Fos protein family [38] and has a massive impact on OC differentiation and production of soluble mediators in bone erosion [39].

Intracellular colocalization and interaction of NaPPS with c-Jun transcriptional factor were observed in this study by immunofluorescence assay emphasizing that the site of action of drug of interest. Binding of c-Fos to the NFATc1 promoter is important for its activation [40]. Suppression of NFATc1 by NaPPS is the consequence of the down-regulation of c-Fos, with the subsequent down-regulation of AP-1 activity and attenuation of OC–specific gene expression required for efficient OC differentiation and bone resorption. Further extension of the study up to detailed work by evaluating specific binding affinity of NaPPS with specific protein at nuclear, sub nuclear domain or nuclear speckles in OC would be much awarded the NaPPS as therapeutic perspective.

Outcome of the present study confirmed that the inhibitory effect of higher concentrations of NaPPS on canine OC differentiation and function, while additional investigations would be required to clarify the mechanism of action of NaPPS on OC in more detail. Although the impact of NaPPS on cell signaling pathways of chondrocytes were well-recognized, invasion in to the OC and its intracellular reactions, competence of osteoclastogenesis from stem cells and effect on functioning structural formation (actin ring) and transcriptional factors have not been considered until probing by this study. To our knowledge, this is the first study to demonstrate that the inhibitory effect of NaPPS on canine bone marrow-derived OC differentiation and bone resorption.

## Conclusions

In this study, we examined the inhibitory effect of NaPPS on in vitro cultured canine OC differentiation and function stimulated by RANKL and M-CSF. Our findings provide useful preliminary information on the concentration of this drug and should help increase understanding and awareness of the opportunity and or limitation of its therapeutic use among dogs. In particular, the inhibitory effects of NaPPS on CTK, MMP-9, NFATc1, c-Fos and AP-1in OC could translate to it beneficial effects in the prevention of osteoporosis and other bone-erosive diseases such as rheumatoid arthritis and bone diseases associated with excessive bone resorption. Furthermore, our results would be a flat form and promising launch for further investigation to identify the intracellular acting sites of NaPPS and more detailed protein interactions for detailed therapeutic mechanism of action.

## Abbreviations

DMOADs: Disease -modifying osteoarthritis drugs
M-CSF: Macrophage colony-stimulating factor
NaPPS: Sodium pentosan polysulfate
OC: Osteoclast
RA: Rheumatoid arthritis
RANKL: Receptor activator of NF kappa B ligand
TRAP: Tartrate-resistant acid phosphatase
